# Computational System Level Approaches for Discerning Reciprocal Regulation of IL10 and IL12 in Leishmaniasis

**DOI:** 10.3389/fgene.2021.784664

**Published:** 2022-01-19

**Authors:** Shweta Khandibharad, Shailza Singh

**Affiliations:** National Centre for Cell Science, SP Pune University Campus, Pune, India

**Keywords:** IL10, IL12, NFAT, leishmaniasis, systems biology

## Abstract

IL12 and IL10 are two of the major cytokines which control the fate of Leishmaniasis. This paper presents two models healthy state and diseased state which shows how secretion of IL12 is responsible for parasite elimination and IL10 can jeopardize the parasite elimination and promote its survival. Epigenetic modification in the host IL12 and IL10 promoter can decide the fate of parasites. It was observed that reciprocal relationship exists between IL12 and IL10 and that is majorly controlled by a transcription factor NFAT5 from Rel family of transcription factors. By targeting this transcription factor at the cellular level, it might be possible to modulate the release of powerful pro-inflammatory cytokines, thereby reducing parasite survival. The mathematical models developed here serves as a step towards finding a key component that can pave a way for therapeutic investigation.

## Introduction

Leishmaniasis is one of the major tropical and subtropical diseases caused by *Leishmania spp* which is a digenetic and dimorphic parasite residing inside the mammalian host. This disease is caused by female sand flies of *Phlebotomus* spp. and *Lutzomyia* spp. by bite and blood meal. Leishmaniasis is a disease that has been classified as an endemic throughout the world, affecting Asia, Africa, the Americas, and the Mediterranean region (Torres-Guerrero et al., 2017). In India, states such as Kerala, Madhya Pradesh, Haryana, Uttarakhand, Himachal Pradesh, Jammu, and Kashmir, Punjab, Assam, Delhi, Bihar, Rajasthan, Jharkhand, Uttar Pradesh and West Bengal has been majorly affected (Thakur et al., 2018) (Kumar et al., 2020). Over 90% of potentially fatal infections occur in these six countries viz., Brazil, Ethiopia, Sudan, South Sudan, India, and Bangladesh (Torres-Guerrero et al., 2017).

Broadly Leishmaniasis is classified into three forms viz., Cutaneous Leishmaniasis (CL), caused by Old World *Leishmania spp* such as *Leishmania major/L. tropica* and is characterized by the appearance of a variety of skin lesions, many of which are innocuous and self-healing. Mucocutaneous Leishmaniasis (MCL) is a long-term disease caused by New World *Leishmania spp*., such as *L. braziliensis*, that can cause extensive facial disfigurement and tissue destruction in the mouth and nose. The vast majority of MCL cases are not fatal. Complications associated with secondary infections, on the other hand, may result in death. Visceral Leishmaniasis (VL) infection caused by *L. donovani*/*L. infantum* is far more severe. It is frequently fatal if left untreated. It is caused by a systemic and progressive infection of macrophages in the reticulo-endothelial systems or lymphoid organs, primarily in the spleen, liver and bone marrow (Karunaweera and Ferreira, 2018).

*Leishmania* parasite has a digenetic life cycle completing in two stages. First, in the *Phlebotomus* sandfly vector and second in Mammalian host. When a sand fly takes a blood meal, it injects metacyclic promastigotes at the bite site. This stage of parasite is the infective stage with morphology characterized by slender shape and is elongated, motile and present extracellularly. Neutrophils are the first cells to be recruited at the bite site. Neutrophils engulf these promastigotes, but due to their short lifespan, they undergo apoptosis and are thus proposed to act as “Trojan horses” used by parasites to hitchhike ride in order to silently enter macrophages, avoiding cell activation (Regli et al., 2017). Inside the macrophage they divide and transform into amastigotes which infect the neighboring tissues. These amastigotes are small, circular and non-motile. When the sand fly takes blood meal from the infected individual, the infected macrophages are also taken up which burst and release amastigotes in the midgut of the sand fly. Later they mature and migrate to proboscis where they can infect another individual (Leta et al., 2014).

One of the prime inflammatory responses after infection is by our innate immunity. The parasite interacts with TLR2 which dimerizes with TLR6 and activates the MyD88 pathway. Through this pathway NFκB activates transcription of pro-inflammatory cytokines such as IL12, IFNγ, TNFα (Soni et al., 2016). These cytokines promotes the synthesis of Nitric oxide (NO) by iNOS which is directly involved in eliminating parasite by killing intracellular parasites within the NO-producing cell as well as those in bystander cells via diffusion across cell membranes (Scott and Novais, 2016). The response of pro-inflammatory cytokines is majorly controlled by IL10 as it is anti-inflammatory cytokine and has been reported to suppress the parasite killing and lead to its survival and persistence (Maspi et al., 2016). Hence, it becomes important to study these cytokines in a holistic way to understand the fundamental pathophysiology process of the disease.

Systems biology is a branch of biology that studies the complex mechanisms which underpin biological systems by treating gene, protein, biochemical network, and physiological responses as integrated parts of a larger system (Mc Auley et al., 2015). Being a holistic approach, Systems biology has been used to study various diseases for instance, using publicly available datasets as well as independent patient samples (Sambarey et al., 2017), identified a signature of ten genes that can distinguish tuberculosis from latent tuberculosis and HIV. In order to study COVID-19 and Influenza co-infection (Soni and Singh, 2021) used Systems biology approach and suggested therapeutic intervention points (Du and Elemento, 2015). stated Systems biology approaches are assisting in the understanding of tumour progression mechanisms and the development of more effective cancer therapies. Systems Biology approach versatility has thus created an urge to use it in studying cytokine reciprocity in Leishmaniasis and to identify players controlling this reciprocity to get a point of intervention; whose modulation may help in restoring the balance and shifting the paradigm to the desired phenotype.

Interaction of LPG (surface antigen of parasite) with TLR2 causes dimerization of TLR2 with TLR6 and activates MyD88 pathway leading to production of IL12 mediated by NFκB (Mukherjee et al., 2015). IL12 is known to induce IFNγ signaling through the JAK-STAT4 mediated pathway (Hamza et al., 2010). IFNγ further initiates a signaling cascade mediated by JAK-STAT1 and upregulates IL12 production (de Groen et al., 2015). Many transcription factors come into play for IL12 production such as IRF1, ICSBP, C/EBP and NFAT5 (Ma et al., 2015). IL12p40 promoter has Nucleosome1 whose histone modification is important in order to produce IL12. Rel family proteins can bind at Nucleosome 1 and lead to production of IL12, among which, NFAT5 is also a transcription factor from the same family (Albrecht et al., 2004) (López-Rodríguez et al., 2001). It can not only upregulate IL12 but also induce iNOS and NLRP3 inflammasome production (Buxadé et al., 2012) (Cornut et al., 2020) NFAT5 is activated by Brx mediated p38 pathway that often functions when the osmolarity of the cell increases, a phenomena which happens during inflammation and also in case of Leishmaniasis (Kino et al., 2009) (Bogdan, 2020). NFAT5 can not only promote parasite killing but it can bind at Sp1 binding site at IL10 promoter and inhibit its transcription (Choi et al., 2016).

Among all the transcription factors, NFAT5 drew our attention as it’s a transcription factor from the Rel family (Albrecht et al., 2004) (López-Rodríguez et al., 2001). TLR sensitive genes are activated by NFAT5. It does so by increasing the production of pro-inflammatory cytokines including IL6 and TNFα by recruiting NFκB and c-fos to their promoter regions. NFAT5 promotes demethylation of H3K27me3 marks from promoter region of pro-inflammatory genes and facilitates recruitment of other transcription factors such as NFκB and enhancers to accentuate their transcription. Sp1 is known to play a central role in IL10 expression (Brightbill et al., 2000; Lunazzi et al., 2021). Its binding at IL10 promoter occurs when H3 phosphorylation on Ser10 at Nucleosome2 takes place (Zhang et al., 2006). It lead us to the assumption and understanding that NFAT5 may induce chromatin remodeling at Nucleosome2 at IL10 promoter that consist of Sp1 binding site so that accessibility of Sp1 to IL10 Nucleosome2 region is hindered and IL10 transcription is hampered (Choi et al., 2016).

Parasite is notorious in nature. It secretes unknown effector molecules which induces chromatin remodeling in the host DNA (Afrin et al., 2019). The cell starts producing IL10 through ERK pathway and upon IL10 signaling the production of HDAC3 and NFIL3 (known IL12 inhibitors) increases leading to suppression of pro-inflammatory cytokine secretion and parasite survival (Yang et al., 2007) (Ma et al., 2015). Parasite also induces SHP-1 which inhibits NFAT5 by dephosphorylating it and thereby hampering parasite clearance and upregulating IL10 production (Zhou et al., 2014).

Using System’s Biology approach, we showed that NFAT5 can be an important transcription factor which can control IL10 and IL12 reciprocity at spatio-temporal level. It may do so by inducing chromatin remodeling at IL12p40 promoter and binding at Nucleosome1 region to upregulate IL12 and inhibiting IL10 synthesis by binding at Sp1 binding site on IL10 promoter and therefore inhibiting anti-inflammatory response. Thus, its presence/absence can play a vital role in deciding the fate of the disease. We also present NFAT5 as an essential component that can lead to parasite clearance by controlling the other determining parasite clearance machinery.

## Materials and Methods

### Reconstruction of Mathematical Models

Signaling pathways allow cells to detect changes in their surroundings, integrate external and internal signals, and respond to them through changes in transcriptional activity, metabolism or other regulatory measures. The function of these cells is critical because it contributes to cell survival and differentiation in various environments. It also determines cell plasticity in multicellular organisms (Klipp and Liebermeister, 2006). In order to understand the complex behavior of signaling networks, we have adopted computational systems modeling approaches that help in deciphering prototypic signaling networks by reconstructing abstract models which emphasize some key features of signaling pathways. This outlook allowed for 1) a systemic examination of all potential input/output relationships, 2) a quantitative assessment of network crosstalk 3) an indicator of signaling network redundancy and 4) the participation of reactions in signaling pathways (Min Lee et al., 2008). In this study, two models were reconstructed; one showing the parasite eliminating phenotype of macrophages, where pro-inflammatory cytokines are expressed (referred as Healthy state), leading to parasite clearance, and the other showing early expression of IL10 (referred as Diseased state) a few hours after *L.major* infection.

For reconstruction of the above signaling networks MATLAB Simbiology toolbox (7.11.1.866) and (9.9.0.1467703) (The Mathworks Inc.) which relies on a XML based representation format Systems biology markup language (SBML) was used.

### Quantitative Modeling of the Biological System

The quantitative modeling of a biological reaction includes kinetics rate laws, parameter estimation and initial component concentration. The following kinetic rate laws are used to define reactions of reconstructed models in the SimBiology toolbox:

1) Law of Mass Action (for association and dissociation reactions),
2) Henri Michaelis Menten equation (for phosphorylation, dephosphorylation and ubiquitination reactions),
3) Hills Kinetic equation (for gene expression reactions).

Preliminary concentrations of the components were determined by taking into account experimentally known concentrations from the literature, which suggests that a cell can secrete 10^3^—10^6^ signaling molecules (Moran et al., 2010).

The model equation was determined by parameter estimation in such a way that the mathematical model mimicked the behaviour of experimentally available data. Mathematical simulation of the defined reactions was done using Stiff Deterministic ODE15s solver (SimBiology toolbox).


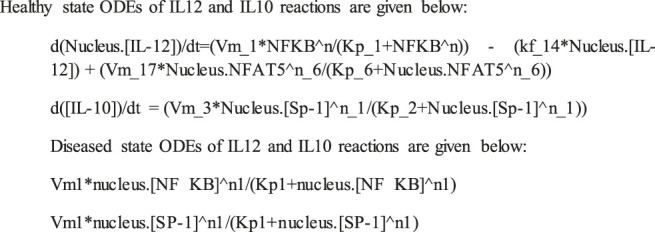


### Sensitivity Analysis

Sensitivity analysis is a tool for determining how a change in parameters affects the system’s behavior. It provides data on the most important parameters that have the greatest influence on system output. Each parameter is linked to a specific biochemical process. As a result, sensitivity analysis may provide insights into how biological experiments should be planned in order to obtain the most pertinent information (Kardynska and Smieja, 2017).

Sensitivities were calculated in a time-dependent manner in relation to the initial concentrations and parameters of each component in the respective model system. SUNDIALS were used as the default solver for the SimBiology toolbox to perform sensitivity analysis, which calculates sensitivity by integrating a model’s original Ordinary Differential Equations (ODE) with the auxiliary differential equations (Nestorov, 1999) (Soni et al., 2018).

### Principal Component Analysis

Principal component analysis (PCA) is a well-known tool for reducing the complexity of multivariate data by filtering out noise and extracting critical components. If we remove those components from the network, the entire network may collapse signifying the principal components are critical in defining the system’s phenotype (Mol et al., 2014; Soni et al., 2018). In nutshell, this method entails creating a scaled sensitivity coefficient matrix S (and its transpose ST) whose elements are Wij derived from the sensitivity analysis (Liu et al., 2005). (score coefficient) = princomp(1) is the MATLAB function used to calculate PCA score, where ‘a’ denotes the m*n matrix of sensitivity coefficients of each component of the reconstructed signaling network (Mol et al., 2014; Soni et al., 2018).

### Flux Analysis and Model Reduction

Flux Analysis is a constraint-based approach that attempts to derive a phenotype for the reactions in a given biological system in the form of a steady-state flux distribution. It is based on the principle that all expressed phenotypes of a given biological system must satisfy fundamental constraints imposed on all cell functions (Min Lee et al., 2008). Copasi (4.8.35), a biochemical network simulator, was used to calculate flux. It solves the ODE of mathematical models and defines the flux of every reaction in the reconstructed biological network.

We combined flux analysis and sensitivity analysis with the goal of developing a systematic method for eliminating reactions that do not contribute significantly to network output. The reason behind this is that reactions deleted during model reduction must have a low sensitivity as well as a low flux. The primary goal of model reduction entails calculating sensitivity coefficients for each component and the flux of the specific reaction based on the concentration of components in a time dependent manner (Liu et al., 2005). Model reduction is useful for reducing network complexity, eliminating spurious parameters and reactions and governing a stable steady state in the network without exhibiting any transient behavior.

### Crosstalk Point

A crosstalk point is a connection between the reconstructed biological signaling network’s pathways. These connections or components define the dynamics of the signaling network and as a result, can serve as pivotal points for network regulation. When specific signaling components are shared between two or more signaling pathways, crosstalk is direct (Vert and Chory, 2011). In this study, the cross talk point for the reconstructed signaling network was calculated by subtracting the degree of a node in the entire network from the degree of that node in an individual pathway and obtaining a non-zero value (Zielinski et al., 2009). Here in this study the crosstalk point was calculated between the TLR signaling, IL12, IFNγ and IL10 pathway.

## Results

### Mathematical Models and Simulation

We demonstrated in the IL12 activation model that LPG binds to TLRs and initiates MyD88-mediated signaling, which causes NFκB to transcribe IL12. In turn, IL12 activates IFNγ, which is responsible for Nitric oxide production and thus parasite clearance. When there is inflammation at the bite site, osmolarity of the cell increases, leading to p38 pathway-mediated activation of a REL family transcription factor NFAT5, which has been reported to induce pro-inflammatory cytokines, Nitric oxide and to suppress IL10 expression.

In the IL10 activation model, we found that the concentration of IL10 had increased with time and IL12 expression had decreased thus leading to parasite survival.

**FIGURE 1:**
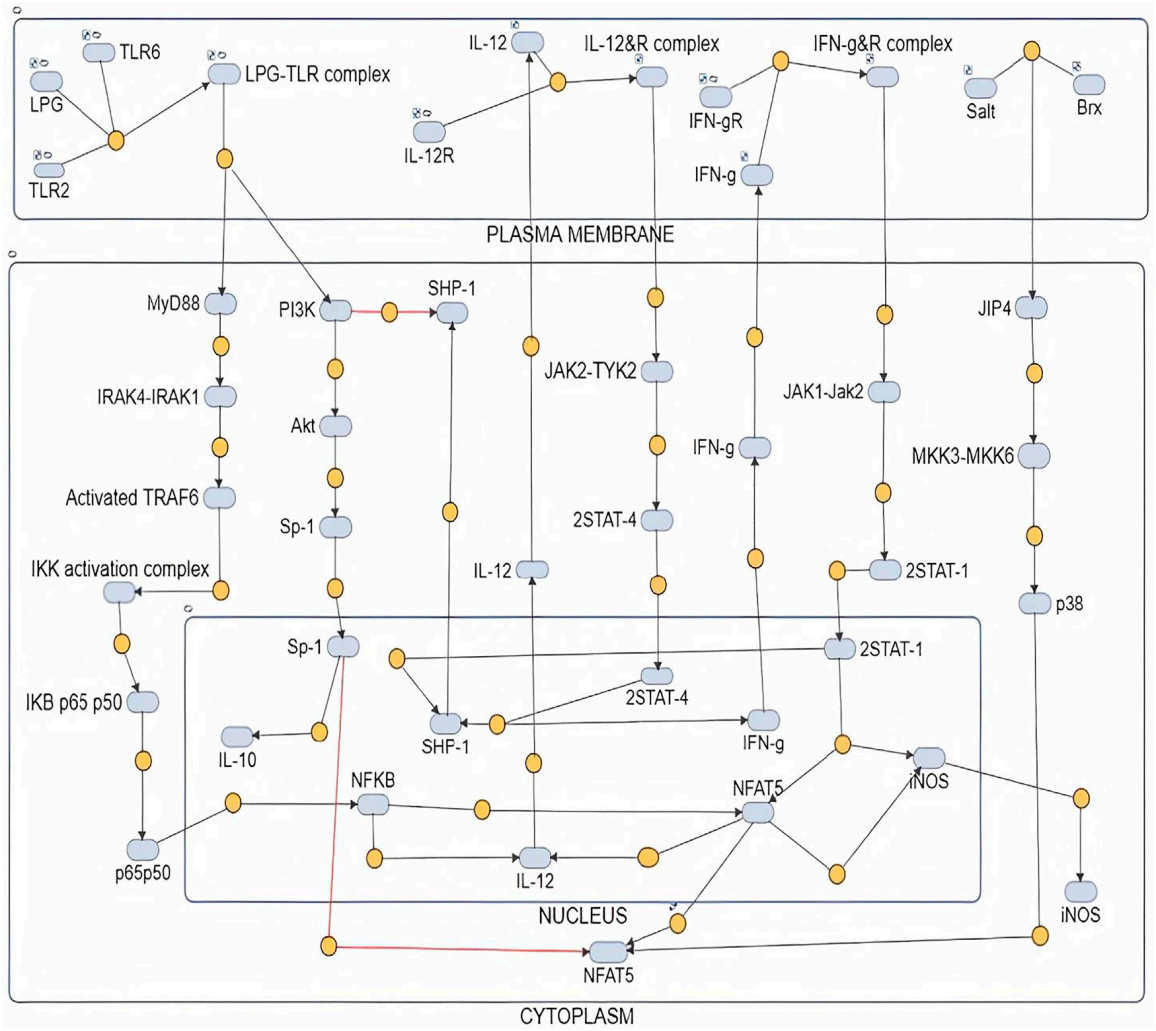
Mathematical model of Healthy state where IL12 is regulating IL10 and leading to parasite clearance.

**FIGURE 2:**
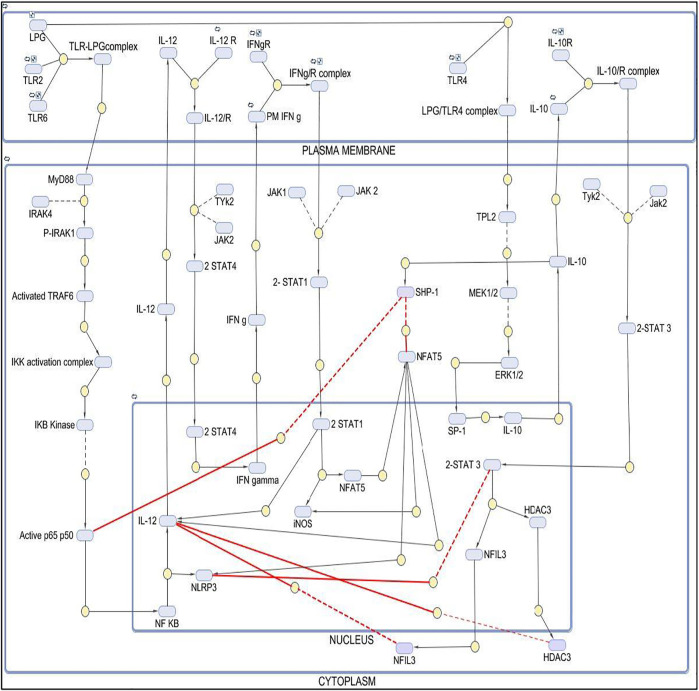
Mathematical model of Diseased state where IL10 is regulating IL12 and leading to parasite survival.

The mathematical models reconstructed consisted of 3 Compartments- Plasma Membrane, Cytoplasm and Nucleus (Figures 1, 2). To obtain the desired behavior in graphical form, the entire network was simulated in a 15s ODE (Ordinary Differential Equation) solver for 20 Unit (Time) and 50 Unit (Time) respectively. Both the pathways have a common starting point from where the signal transduction initiates and that is when the parasite interacts with TLR2-TLR6 to activate MyD88 mediated NFκB signaling cascade which leads to downstream phenotype conferring events.

#### Healthy Model

**FIGURE 3:**
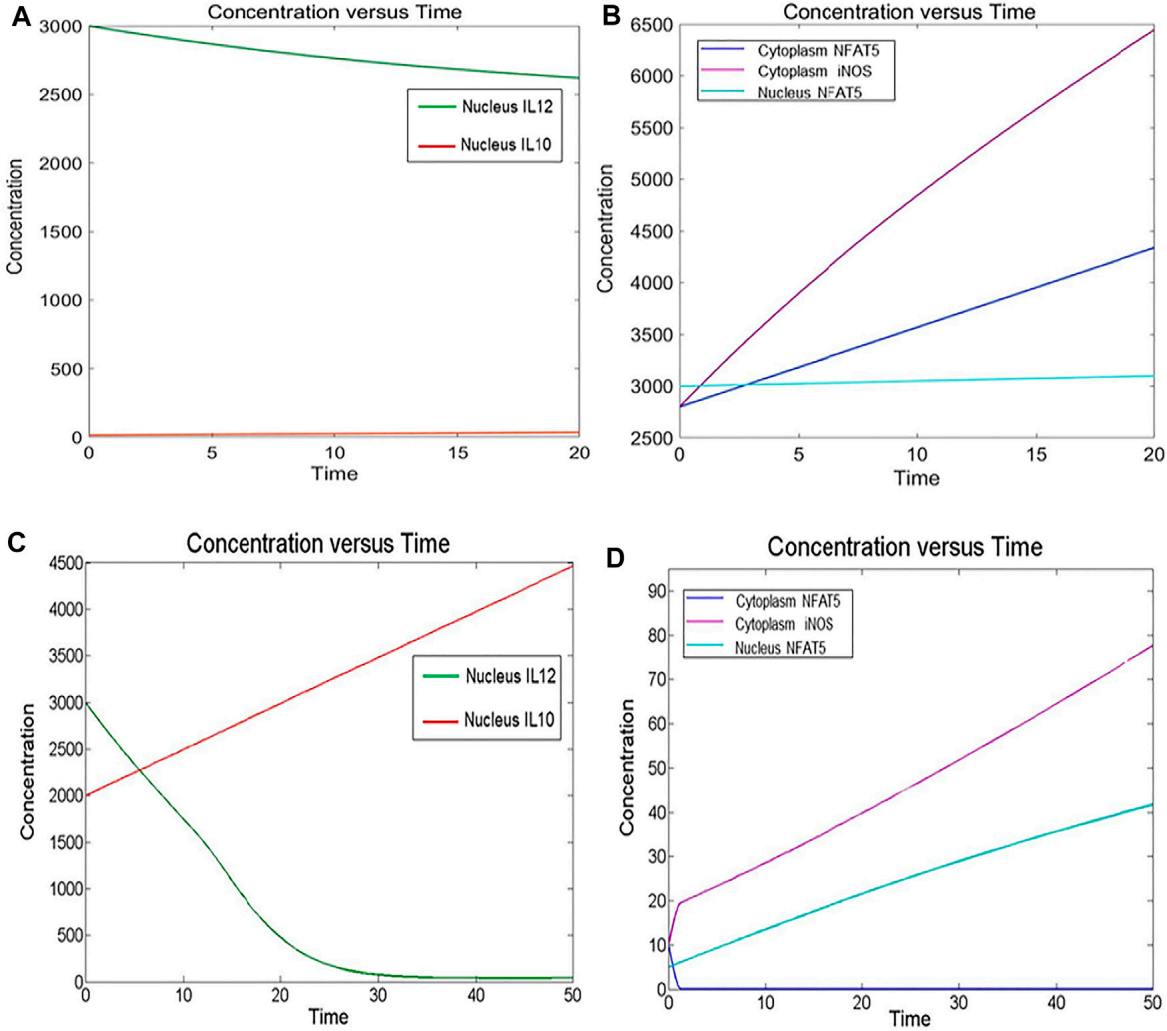
Simulation of healthy state and disease state models: **(A)** Healthy state showing concentration of IL12 and IL10 for 20Unit Time. **(B)** Healthy state showing concentration of NFAT5 and iNOS for 20Unit Time. **(C)** Diseased state showing concentration of IL12 and IL10 for 50Unit Time. **(D)** Diseased state showing concentration of NFAT5 and iNOS for 50Unit Time.

The mathematical model has three compartments namely membrane, cytosol, nucleus. There are a total 43 signaling components, 114 parameters, and 40 reactions. The simulation was performed for 20 time units, using Stiff Deterministic ODE15s solver (SimBiology toolbox) which generates the first order non-linear ODEs for each reaction in the system. We observed that the major outputs by the system were the production of iNOS, NFAT5 and IL12 (Figures 3A,B). The model was submitted to the Biomodel database with identifier MODEL109080001.

#### Diseased Model

The mathematical model has three compartments namely membrane, cytosol, nucleus. There are a total 70 signaling components, 83 parameters, and 46 reactions. The simulation was performed for 50 time units, using Stiff Deterministic ODE15s solver (SimBiology toolbox) which generates the first order non-linear ODEs for each reaction in the system. We observed that the major outputs by the system were the production of IL-10 with decrease in iNOS, IL12 and NFAT5 (Figures 3C,D). The model was submitted to the Biomodel database with identifier MODEL201204000.

By observing simulation results for both the models, we can see the behavior of IL12, IL10, NFAT5 and iNOS change over the period of time. In the healthy state concentrations of IL12, NFAT5 and iNOS is more and IL10 concentration is lesser which suggests that the model phenotype is leading towards parasite clearance (Figures 3A,B). But, as the time progresses concentration of IL10 increases and concentrations of IL12, NFAT5 and iNOS decreases suggesting that the parasite survival may be happening (Figures 3C,D).

**FIGURE 4:**
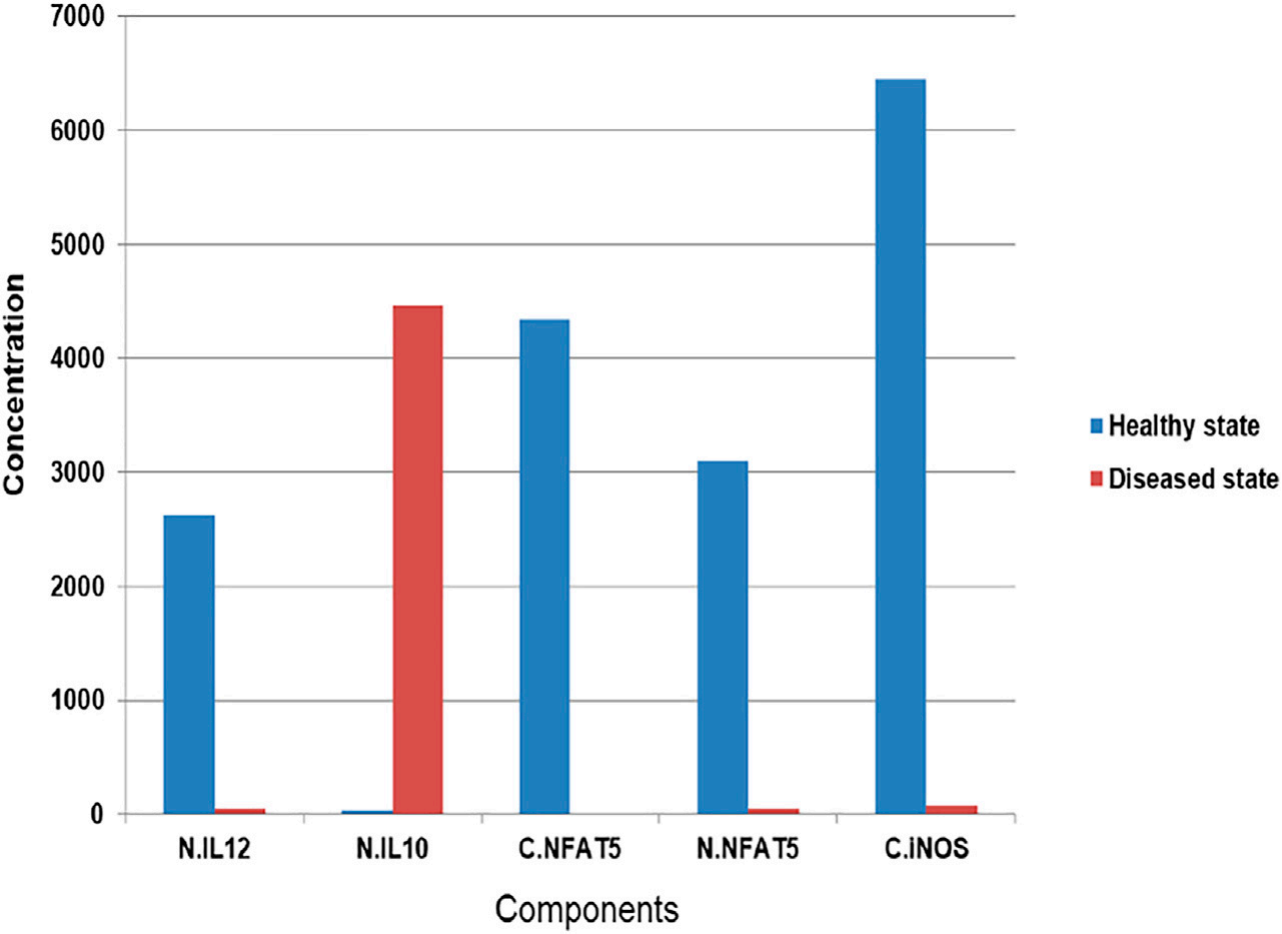
Concentration of components after simulation in Healthy as well as Diseased state.

By observing both the models we may say that the expression levels of IL12 and IL10 can play a major role in determining the phenotypic state of the *Leishmania spp* infected cells (Figure 4).

## Principal Component Analysis

**FIGURE 5:**
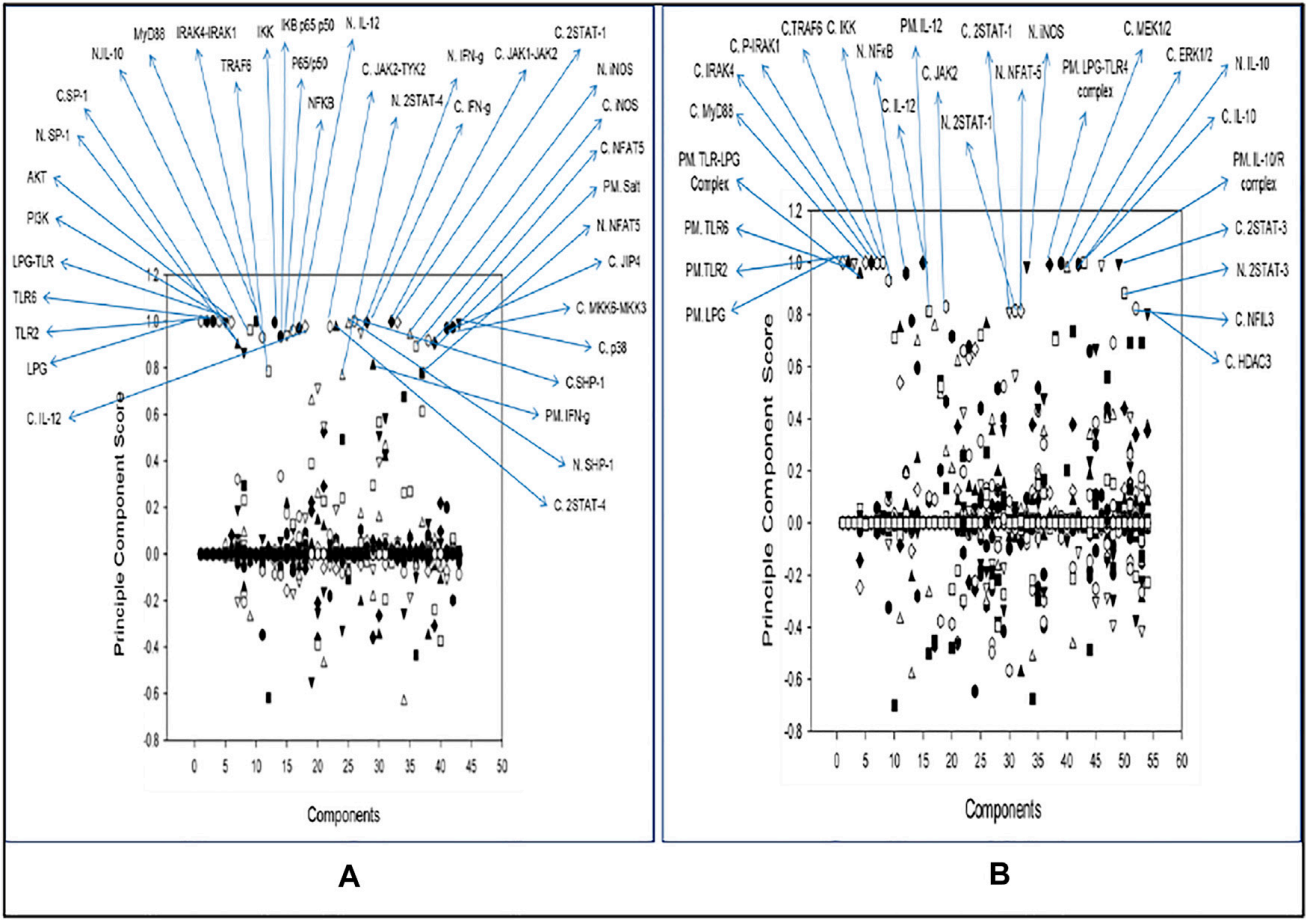
Principal Component Analysis: **(A)** Healthy state. **(B)** Diseased state.

Common components in both the pathways after PCA were LPG, TLR2, TLR6, LPG-TLR2 and TLR6 complex, MyD88, IRAK4, IRAK1, TRAF6, IKK activation complex, NFκB, IL12, JAK2, 2STAT-1, NFAT5, iNOS, and IL10 (Figure 5). Other than the common components there were many other molecules which were model specific but also crucial as PCA allows identification and increase in the interpretability of critical components by minimizing information loss. Unique components which stood out from comparative analysis of both the model were NFIL3 and HDAC3 as they were specific to disease state model. IL10 can target NFIL3 to bind 10 kb upstream of the IL12p40 transcriptional start site and inhibit its synthesis. HDAC3 is involved in IL-10-induced histone deacetylation of the IL12p40 promoter (Ma X et al., 2015). The list of all the Principal components with their score is mentioned in Supplementary Material S2, S6.

Flux Analysis: As flux majorly contributes to the model phenotype; we observed following reactions had high flux:

According to the comparative flux analysis, the reaction associated with Healthy state model which have high flux are cytokines producing reactions such as IL12, IFNγ, osmolarity inducing reactions and iNOS inducing reaction. The reactions associated with diseased state which have high flux are inhibitory products of NFAT5 and IL12 suggestive of suppressing their synthesis. Hence, these high flux reactions can be associated with polarizing the model to healthy and diseased phenotype.

**FIGURE 6:**
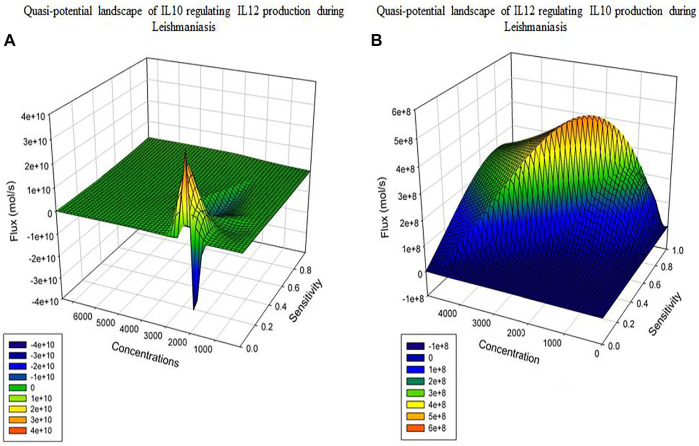
3D representation of reduced Model: **(A)** Healthy state **(B)** Diseased state.

Model Reduction: For both the models we can observe the Quasi-potential landscapes (Figure 6). Both the graphs show us a dome shaped pattern or a peak like plot which shows the distribution of the model in the form of high PC, flux and components present at a higher concentration to fall at the top of the dome. As we move down the dome and peak; low PC, flux and concentration components will be present. As per PCA and flux; the reactions which contribute towards network output are the phenotype determinants.

### Cross Talk Point

**FIGURE 7:**
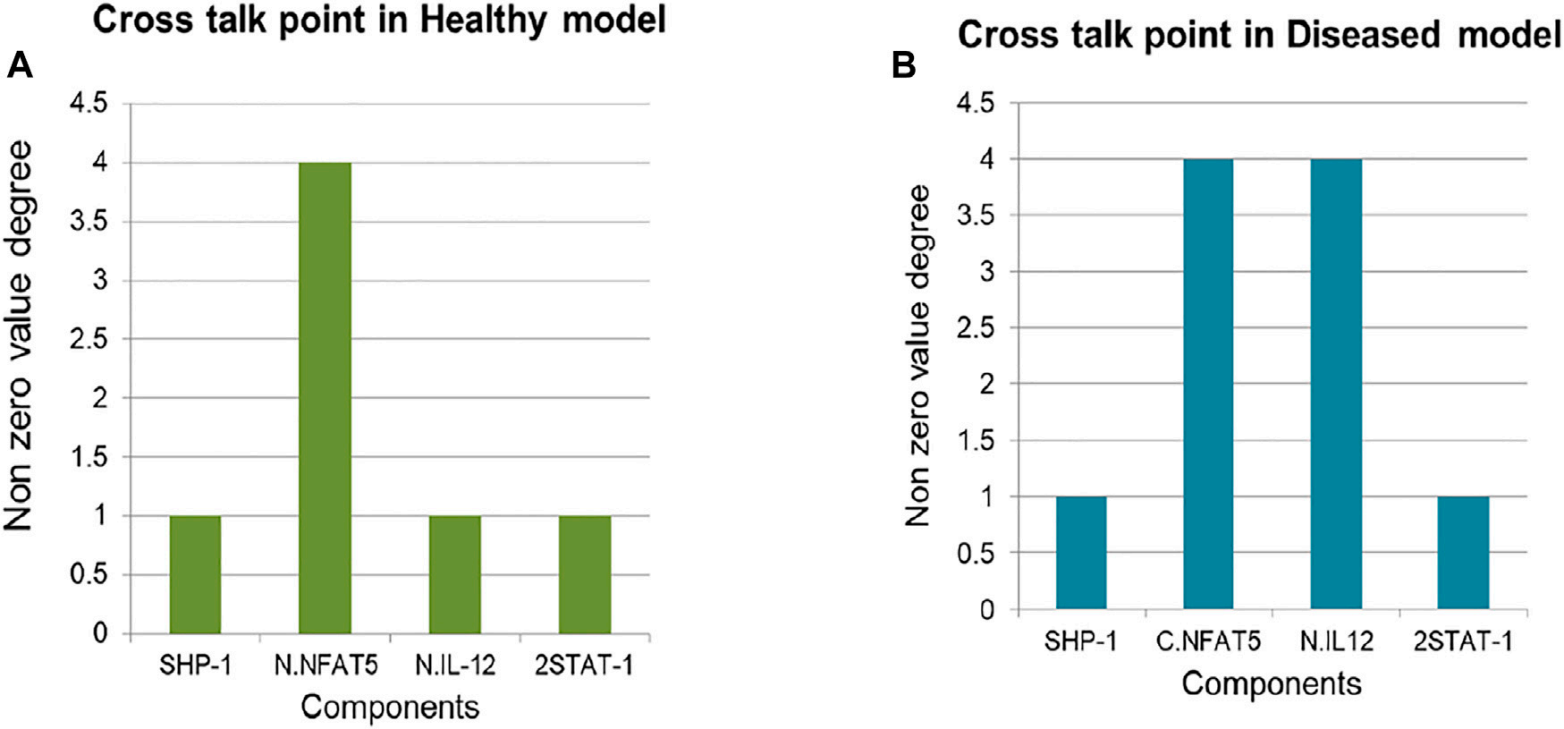
Cross talk point identification: **(A)** Healthy state **(B)** Diseased state.

In both the models 4 cross talk points were identified: Nucleus IL12, NFAT5, 2STAT-1 and SHP-1. Out of which Nucleus IL12 had crosstalk value of 1 and 4, NFAT5 had 4 and 4, 2STAT-1 had 1 and 1 and SHP-1 had 1 and 1 in Healthy state and Diseased state respectively (Figure 7).

## Discussion

Changes in the cytokines level in macrophages decide the fate of the disease. Reciprocity of cytokines have been previously investigated for IFN-γ and IL4 where, IFN-γ regulated Th2 response and IL4 regulated Th1 response during Leishmaniasis in murine models at temporal levels (KEMP et al., 1993). In murine leishmaniasis, IFN- *γ* and IL-4 are produced reciprocally in resolving or progressive infection, supporting the hypothesis that distinct T-helper subsets moderate the spectrum of this infectious disease (Heinzel et al., 1989). Moreover, *L. donovani* uses specific histone lysine methyltransferases/demethylases to redirect epigenetic programming of M(LPS + IFN-γ)/M(IL-10) genes for successful host establishment (Parmar et al., 2020). Hence, epigenetics have created an arena for regulating reciprocity between different cytokines, which makes cytokine reciprocity an enticing domain to study the remodeling of the immune response.

**FIGURE 8:**
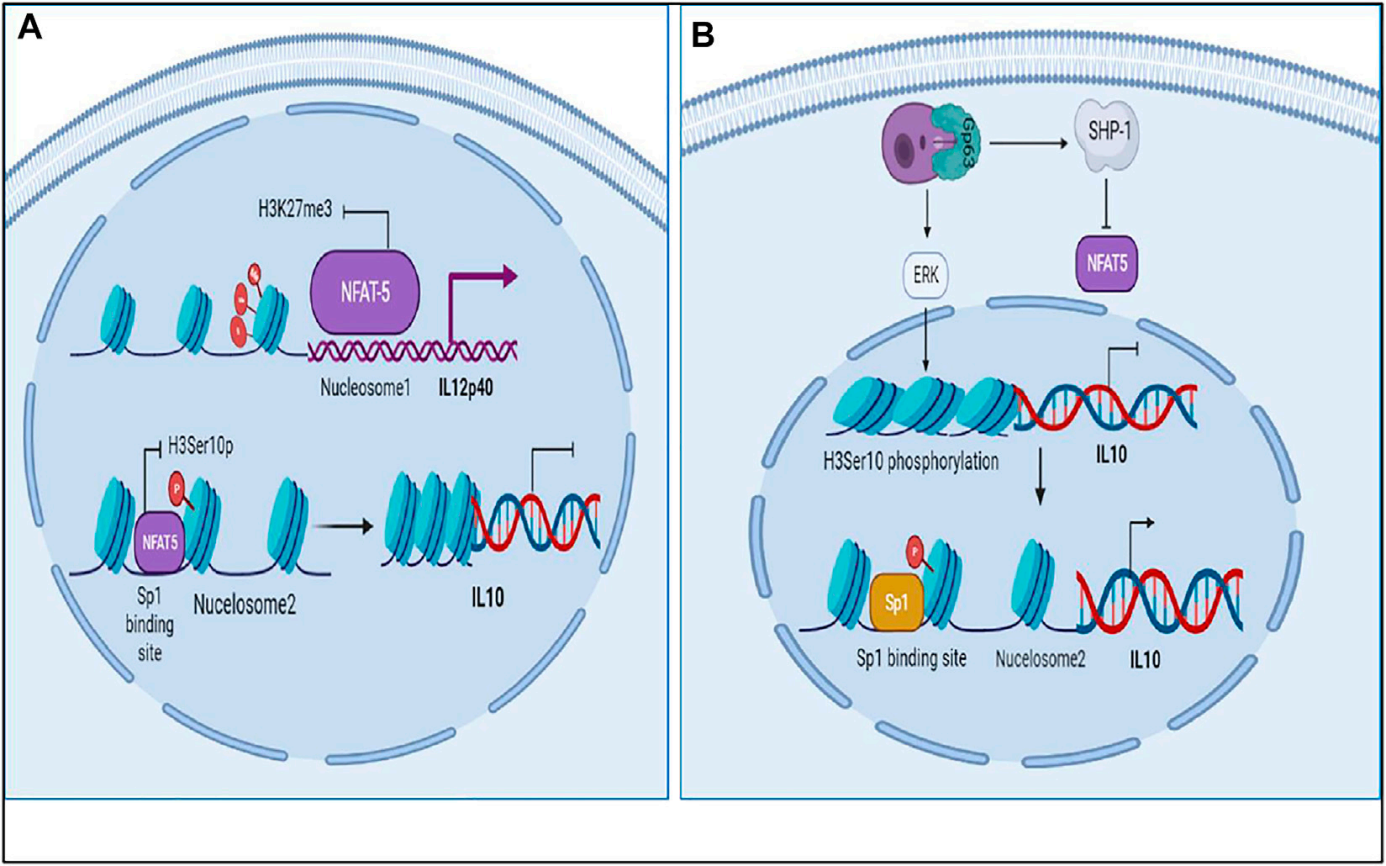
Epigenetic regulation of IL12 and IL10 by NFAT5 in Leishmaniasis: **(A)** NFAT5 regulating IL12 and IL10 synthesis by controlling Histone modifications at IL12 and IL10 nucleosomes at promoter region. **(B)** NFAT5 being regulated by SHP-1 and parasite promoting IL10 production by directing epigenetic remodeling.

**FIGURE 9:**
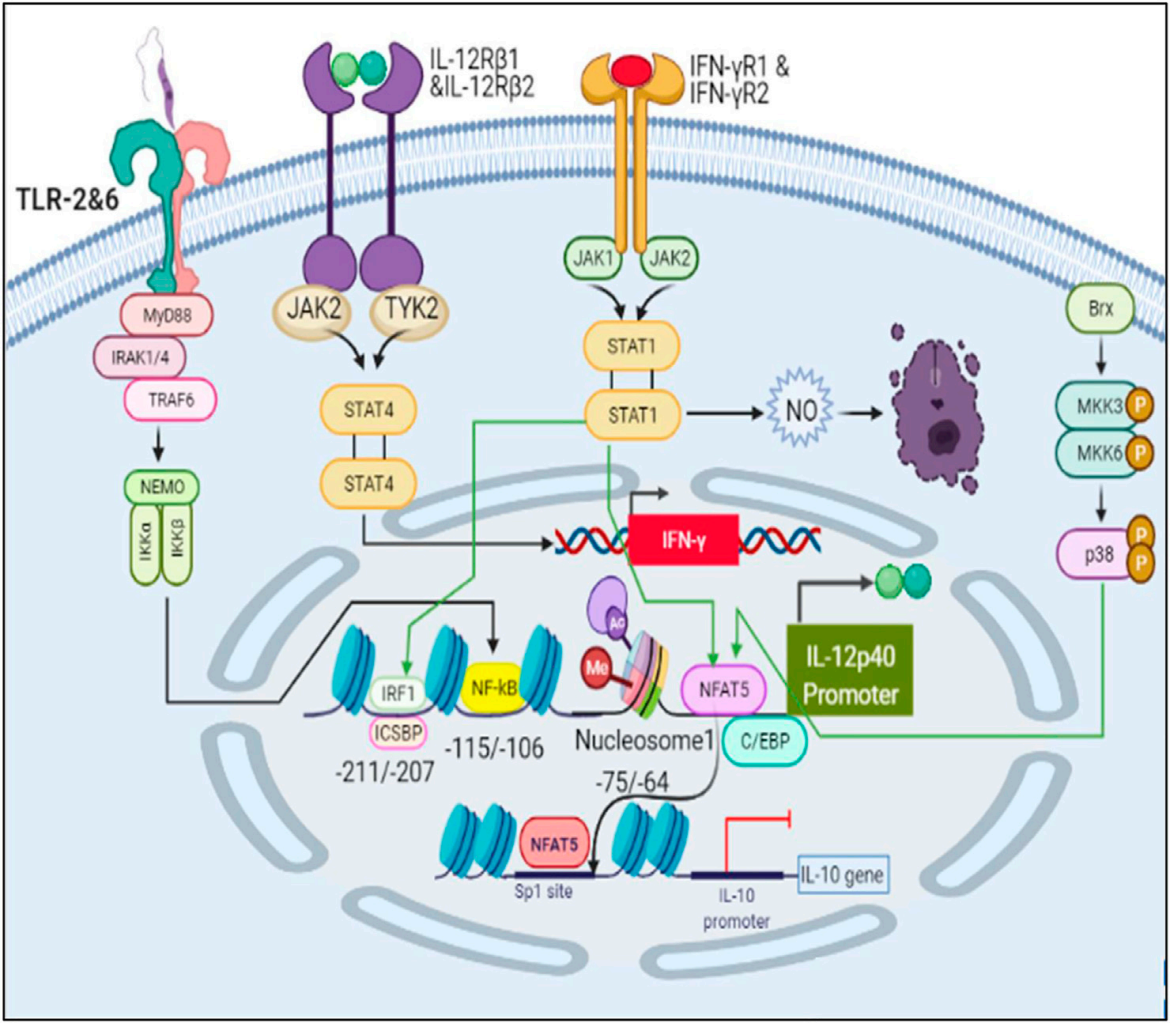
Schematic representation of IL12 production and IL10 inhibition leading to parasite clearance.

**FIGURE 10:**
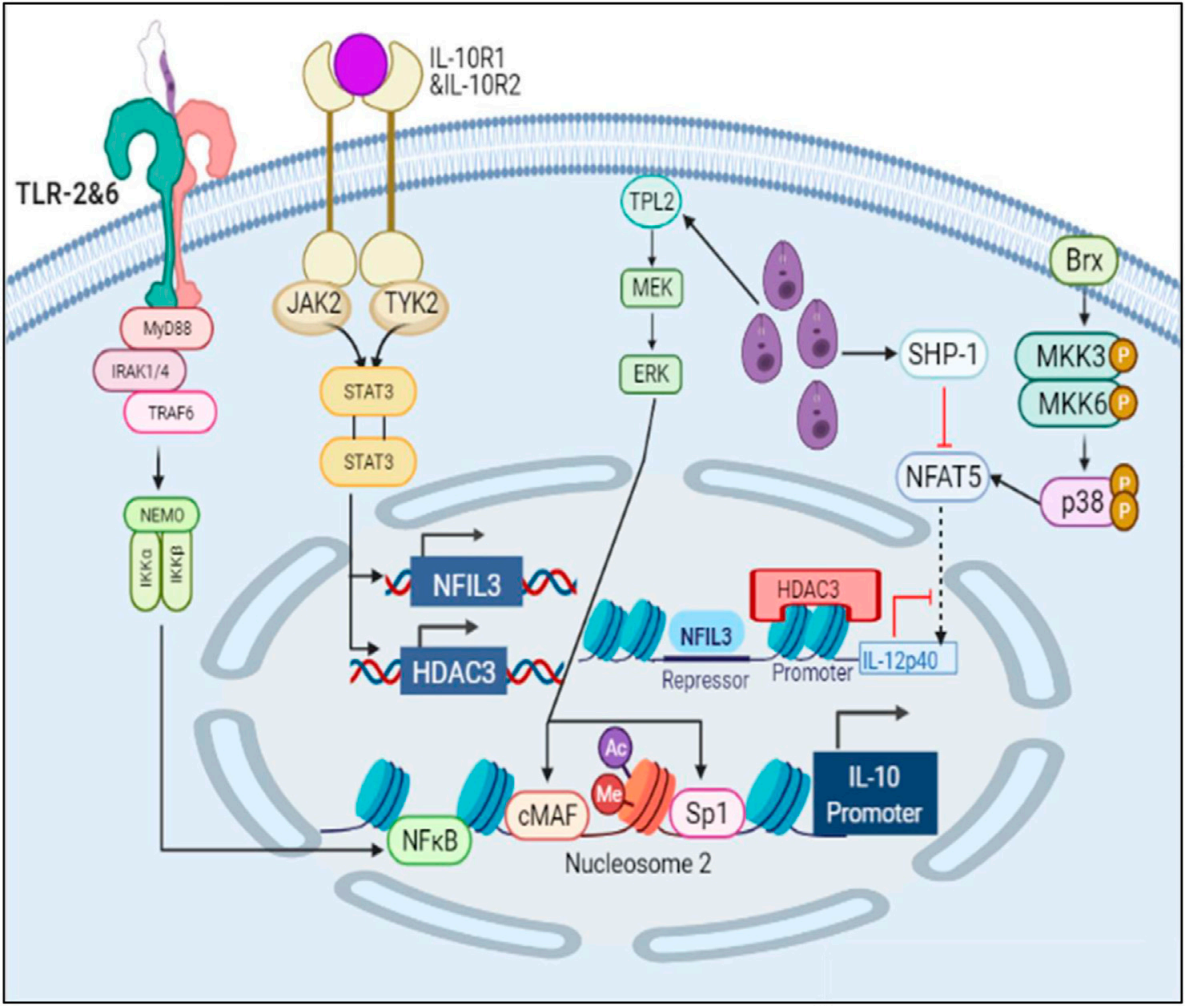
Schematic representation of IL10 production and IL12 inhibition leading to parasite survival.

The results suggest that as the levels of IL12 decreases and IL10 increases the phenotype of the macrophage changes and that hampers the NO secretion which correspondingly inhibits parasite clearance and infectivity. Through our mathematical models we observed that when IL12 levels are high during the initial response, IL10 levels were low which promoted iNOS secretion and parasite clearance but as the time progressed the parasite promotes changes in the macrophages which hampers IL12 secretion and promotes IL10 secretion. As the secretion of IL10 increases, the levels of IL12, NFAT5 and iNOS decreases suggesting that parasite survival machinery has been activated (Figures 1-3). As, epigenetic regulation of host chromatin structure is influenced by the parasite (Afrin et al., 2019), our data shows that NFAT5 can be a key regulator of epigenetic modification of cytokines and may play a role in parasite elimination. NFAT5 presumably promotes inhibition of IL10 secretion by inhibiting H3Ser10 phosphorylation that recruits Sp1 on Nucelosome2 at the promoter region and at the same time it promotes demethylation of H3K27me3 marks from promoter region of IL12 at Nucleosome1 and recruits other transcription factors on IL12 promoter axis. These epigenetic modifications can be one of the most important changes responsible for changes in the immune response during Leishmaniasis (Krayem and Lipoldová, 2021). Over the course of time parasite that have skipped the clearance tends to hamper the ability of important transcription factors such as NFAT5, 2STAT-1 to participate in IL12 secretion by regulating them. *Leishmania* parasite is known to activate SHP-1 to dephosphorylate NFAT5 and prevent IL12 secretion. Moreover, parasite for its own survival modulates the host epigenetics by promoting ERK mediated H3 phosphorylation that makes Sp1 binding site again accessible to Sp1 for binding at IL10 promoter region (Yang et al., 2007) (Zheng et al., 2020). Since NFAT5 can now not bind at IL10 promoter by getting dephosphorylated by SHP-1, Sp1 can now access the modified H3 and carry out IL10 transcription (Figure 8). IL10 signaling promotes upregulation of IL12 inhibitors such as HDAC3 and NFIL3 which can now epigenetically and transcriptionally inactivate IL12 synthesis (Figures 9, 10).

Through PCA we tried to figure out key components which help in deciding the phenotype of the infected cell. The components of TLR2 and TLR6 mediated MyD88-NFκB pathway plays an important role in the phenotype decision making as they are known for promoting cytokine signaling (de Veer et al., 2003). Here, we also observed that IL12 and IL10 are principal components and they are of opposing nature hence, their synthesis and secretion levels will directly affect the parasite survival (Kane and Mosser, 2001) (Park et al., 2002). Jak2 is a tyrosine kinase which is recruited to IL12Rβ2, IFNγR2 to help in IL12 and IFNγ signaling (Bacon et al., 1995). iNOS is also a principal component as it is directly participating in parasite clearance by converting free N2 from metabolic pathways to NO (Olekhnovitch and Bousso, 2015). Another enticing aspect which came into the spotlight was transcription factors 2STAT-1 and NFAT5. Dimer form of phosphorylated STAT1 is well known in the literature for its ability to promote transcription of IFNγ (Bhardwaj et al., 2010). It is also known to activate Th1 response against parasites (Kima and Soong, 2013). NFAT5 is a member of the NFAT family of transcription factors which includes NFAT1, NFAT2, NFAT3 and NFAT4. These transcription factors are involved in osmoregulation of cells (Cen et al., 2020). It has been reported that NFAT5 binds to IL12p40 promoter region and accentuates IL12p40 synthesis in response to parasites (Buxadé et al., 2012; Tellechea et al., 2018). Since NFAT5 is also known to bind to the IL10 promoter region where Sp1 binding site is present and hampers IL10 synthesis (Choi et al., 2016), its dual functionality can not only help in increasing pro-inflammatory cytokine response but may also inhibit IL10 secretion so that parasite clearance can take place.

Flux Analysis decides the flow of molecules at a given time (Beguerisse-Díaz et al., 2018). In the healthy state, we observed that the reactions which were promoting IL12 secretion (Table 1) and the reactions that promoted IL10 production had low flux (S3 & S7). In the diseased state, the reactions which promoted IL10 synthesis and IL12 inhibition had high flux (Table 2). Thus the flux and PCA together lead us to figure out important components in the model which are (IL12, IL10, NFAT5, iNOS).

**TABLE 1:** High flux reactions in Healthy state model.

Reactions	Flux (mol/s)
TLR6+TLR2+LPG→LPG-TLR complex	400000
JIP4→ MKK3-MKK6	485.915
N.iNOS → C.iNOS	240
C.IL12→ PM.IL12	232
C. 2STAT-4→ N.2STAT-4	220
N.IL12→ C.IL12	150
C.IFNγ→ PM.IFNγ	118.4
C.2STAT-1→ N.2STAT-1	142
Salt + Brx→ JIP4	140

**TABLE 2:** High flux reactions in Disease state model.

Reactions	Flux (mol/s)
C.IL10→SHP-1	2020.12
N.HDAC3→ C. HDAC3	1506.92
N. NFIL3→ C.NFIL3	1004.41
C.2STAT3 → N.2STAT3	320.31
C.NFIL3 + N. IL12→ C.NFIL3	171.441
N.2STAT3 → N.NFIL3 + N. HDAC3	119.962

Fluxes of other reactions are attached in the Supplementary Table S3, S7.

After performing model reduction the quasi potential landscape of both the models shows a dome and a peak like architecture. The reactions which were of high flux, high concentration and had high sensitivity coefficient lied at the top of the dome and peak whereas, the reactions with lower flux, concentration and sensitivity were at the base of the dome and peak (Figure 6). This materialized the crucial components and demonstrated that the model output or phenotype contributors could be IL12 and IL10 synthesis and secretion reactions, as well as reactions that inhibited their synthesis.

Crosstalk point helps in identification of the components which can connect two or more pathways. IL12 helps in IFNγ synthesis and its secretion is important to maintain healthy phenotype of the cells (Park et al., 2002), suggestive of the fact that for the parasite clearance IL12 is important. 2STAT-1 promotes synthesis of IFNγ as well as iNOS hence, STAT-1 is also promoting IL12 synthesis and promoting parasite clearance through iNOS upregulation (Bhardwaj et al., 2010) (Isnard et al., 2012) (Jayakumar et al., 2008). NFAT5 is not only promoting IL12 synthesis but also inhibiting IL10 (Choi et al., 2016). It also upregulates other processes which promote parasite clearance that is NLRP3 inflammasome production and iNOS secretion (Buxadé et al., 2012) (Cornut et al., 2020). Hence, NFAT5 has roles in promoting and inhibiting different processes which promote parasite survival. SHP-1 is a protein phosphatase which dephosphorylates NFAT5 (Yang et al., 2007) and therefore regulates NFAT5 activity in response to Leishmaniasis.

Through our computational analysis we could infer that NFAT5 may promote epigenetic remodeling in IL12 and IL10 promoter axis where it upregulates IL12 production and decreases IL10 secretion. Simultaneously, over the course of time as the parasite struggles for its survival, it upregulates SHP-1 in a manner that NFAT5 is dephosphorylated and inactivated for loss of its epigenetic modification regulating abilities, thereby changing the cytokines level altogether and apparently parasite survives to infect the other cells. Thus, NFAT5 can be taken as a point of intervention to altogether investigate the parasite clearance regime.

To conclude we posit that computational modeling of IL12 and IL10 reciprocity through NFAT5 in Leishmaniasis helps us to understand the signaling mechanism responsible for parasite survival. Thus, the system’s biological approach provides us with the necessary lead in a specific reconstructed biological network and by modulating NFAT5; we might be able to find a better therapeutic for treating Leishmaniasis.

## Data Availability

The original contributions presented in the study are included in the article/Supplementary Material, further inquiries can be directed to the corresponding author.

## Glossary

C/EBP: CCAAT-enhancer-binding proteins
COVID-19: Coronavirus disease, 2019
HDAC3: Histone deacetylase 3
ICSBP: Interferon consensus sequence-binding protein
IFNγ: Interferon gamma
IL10: Interleukin 10
IL12: Interleukin 12
iNOS: inducible Nitric Oxide synthase
IRAK1: Interleukin 1 Receptor Associated kinase 1
IRAK4: interleukin-1 receptor-associated kinase 4
IRF1: Interferon Regulatory Factor 1
JAK: Janus kinase
LPG: Lipophosphoglycans
MyD88: Myeloid differentiation factor 88
NFAT5: Nuclear Factor Of Activated T Cells 5
NFIL3: Nuclear Factor, Interleukin 3 Regulated
NFκB: Nuclear factor kappa B
NLRP3: NLR family pyrin domain containing 3
p38: p38 mitogen-activated protein kinases
PC: Principal component
SHP-1: Src homology 2 domain-containing protein tyrosine phosphatase 1
2STAT-1: Phosphorylated dimer of Signal transducer and activator of transcription 1
2STAT-4: Phosphorylated dimer of Signal transducer and activator of transcription 4
2STAT-3: Phosphorylated dimer of Signal transducer and activator of transcription 3
STAT1: Signal transducer and activator of transcription 1
STAT3: Signal transducer and activator of transcription 3
STAT4: Signal transducer and activator of transcription 4
TRAF6: TNF Receptor Associated Factor 6
TLR2: Toll Like Receptor 2
TLR6: Toll Like Receptor 6
TNFα: Tumor Necrosis Factor alpha
